# Joint Probability Analysis of Extreme Precipitation and Storm Tide in a Coastal City under Changing Environment

**DOI:** 10.1371/journal.pone.0109341

**Published:** 2014-10-13

**Authors:** Kui Xu, Chao Ma, Jijian Lian, Lingling Bin

**Affiliations:** 1 State Key Laboratory of Hydraulic Engineering Simulation and Safety, Tianjin University, Tianjin, China; 2 College of Water Sciences, Beijing Normal University, Beijing, China; University California Los Angeles, United States of America

## Abstract

Catastrophic flooding resulting from extreme meteorological events has occurred more frequently and drawn great attention in recent years in China. In coastal areas, extreme precipitation and storm tide are both inducing factors of flooding and therefore their joint probability would be critical to determine the flooding risk. The impact of storm tide or changing environment on flooding is ignored or underestimated in the design of drainage systems of today in coastal areas in China. This paper investigates the joint probability of extreme precipitation and storm tide and its change using copula-based models in Fuzhou City. The change point at the year of 1984 detected by Mann-Kendall and Pettitt’s tests divides the extreme precipitation series into two subsequences. For each subsequence the probability of the joint behavior of extreme precipitation and storm tide is estimated by the optimal copula. Results show that the joint probability has increased by more than 300% on average after 1984 (*α* = 0.05). The design joint return period (RP) of extreme precipitation and storm tide is estimated to propose a design standard for future flooding preparedness. For a combination of extreme precipitation and storm tide, the design joint RP has become smaller than before. It implies that flooding would happen more often after 1984, which corresponds with the observation. The study would facilitate understanding the change of flood risk and proposing the adaption measures for coastal areas under a changing environment.

## Introduction

Cities with large population are vulnerable to flooding from extreme weather events [1] and the climate change and urban expansion may increase levels of risk of extreme events in many cities [2]–[6]. Over the past several decades, China has experienced explosive economic growth and phenomenal urbanization. Under this backdrop, an increasing trend of flooding events resulting from extreme precipitation can be observed in recent years [7]. For example, the heaviest rain in 60 years hitting Beijing resulted in huge flooding in July, 2012, claiming the lives of 79 people and causing at least 10 billion Yuan loss. Besides, according to the statistics released by the Ministry of Housing and Urban-Rural Development of the People’s Republic of China (MOHURD), 62 percent of 351 cities in China suffered urban flooding during 2008 to 2010, specifically, among them 137 cities experiencing 3 times. Due to increased flooding events, public awareness of hydro-meteorological extremes has significantly increased and calls for urgent adaptation measures, such as improvement in drainage systems to reduce the risk. In China, coastal cities have overwhelming population density and rapid economic growth [8]. Thus, the adaptation planning will benefit these areas significantly. However, quantifying the risk and understanding the changes in the extreme weather events are challenges in planning adaptation [9]–[10].

In China, the design criterion of drainage facilities in the urban area is designed only by rainfall as heavy rainfall is the direct factor causing local flooding. However, for a coastal city, heavy rainfall and storm tide are both inducing factors of flooding [11]. Runoff of precipitation collected by drainage systems flows directly or is pumped into the sea or the tidal river. Storm tide has an influence on the drainage capability with a worse situation of flow backward, or directly causes coastal flooding. Some evidences also can be found to prove the existed dependence between precipitation and tide level [12]–[14]. Ignoring storm tide’s impact to flooding may lead to underestimation of the design standard of flood defenses and the associated risk. In our previous work [13], the joint impact of rainfall and tide level on flooding risk has been estimated by a hydrodynamic approach, illustrating that rainfall and tide level both have a notable impact on flooding in a coastal city. Therefore, the joint probability of extreme precipitation and storm tide should be proposed in determining of flood preparedness design.

Copulas are being increasingly employed in the analysis of multivariate events [15]–[19]. The most important advantage of using copula is that the marginal properties and dependence structure of the random variables could be investigated separately [20]. In the hydro-meteorological applications, copulas have been widely applied in the analysis of precipitation behavior in recent years. Salvadori and De Michele [21] estimated the dependence between the intensity and duration of storm rainfall. Balistrocchi *et al.* [22] and Bárdossy *et al.* [23] revealed a non-ignorable dependence between rainfall volume and duration. Gyasi-Agyei *et al.* [24] modeled the dependence among the internal structure of rainfall events, such as storm depth and duration. Zhang *et al.* [25] estimated the joint distributions of rainfall intensity and duration, intensity and depth, depth and duration. Wang *et al.* [26] and Zhang *et al.* [27] investigated the joint distribution of intensity, volume and duration of rainfall events. It is obvious to find out that the focus about rainfall events using copula is much on the characteristics of rainfall itself, i.e. on the dependence among precipitation intensity, duration, and volume. To object, only limited amount of work has been carried out to employ copulas to estimate the dependence of rainfall and tide level. Archetti *et al.* [12] estimated the correlation between rainfall and tide level in term of the 69 rain events throughout year 2009 in the northern area of the Municipality of Rimini. In the previous work [13], we also have estimated the dependence between extreme rainfall and tide level in Fuzhou City. The work in this paper not only focuses on the joint probability of extreme precipitation and storm tide, but also considers the change of the probability.

It is widely accepted that precipitation changes, particularly extremes, are one type of significant perspectives to scientifically evaluate the behaviors and changes of climatic systems [28]–[32]. Nowadays, much more attention has been paid to the internal variability of precipitation itself, such as the temporal and spatial characteristics [33]–[35]. The work in this paper takes into account the precipitation change when we analyze its associated risk.

The purpose of this paper is to investigate the joint probability of extreme precipitation and storm tide to have a better understanding of the increased flood risk in the setting of the changing environment. The study would reveal how the extreme weather events change and why flooding has occurred more often recently in a coastal city of China, and then put forward the design standard of future flooding preparedness as to improve flood risk management. In Section 2, the study area and data are introduced. The methodology, consisting of the copula model and detecting method of precipitation change, is described in Section 3. Section 4 reports the analysis of the joint probability of extremes, and discusses the joint return period (RP) of extreme precipitation and storm tide for flooding preparedness in a coastal city. Section 5 discusses the disadvantage of the traditional design standard of flood defense in China and proposes design standard for flooding preparedness demand in the future. Also, conclusions are given in this section.

## Study Area and Data

The data in urban Fuzhou, a coastal city in the southeast of China, are employed in this study. The urban area of Fuzhou, approximately covering an area of 100 km^2^, is surrounded by mountains on three sides and on another side by a tidal river connected to the East China Sea. According to historical records (1949–2011), Fuzhou was struck by tropical cyclones 56 times, the main sources of heavy rains and storm tide. 24-h precipitation events typically drive local flooding events in urban Fuzhou. Storm tide has a significant influence on flooding, always aggravating the inundation by impeding drainage of flood water. For example, high storm tide brought by Typhoon Longwang in 2005 impeded the discharge of the rain runoff resulting in the inundation of a 13.69 km^2^ area and over 62 people died.

In this study, two steps are carried out to analyze the joint probability of extreme precipitation and storm tide. First, given that the heavy rain is the direct factor to flooding in Fuzhou, the paired observations {*h*, *z*} of 24-h precipitation *H* and highest tide level *Z* during the annual maximum 24-h precipitation events from 1952 to 2009 are chosen to build their joint distribution. Depending on the joint distribution function, the joint probability of any combinations of *H* and *Z* could be estimated, including the extreme values. Then, the depth-RP relationship of precipitation and level-RP relationship of tide level in Fuzhou City are employed to determinate the RP of the extreme values in the first step. Table 1 concludes the relationship of extreme values and their RPs. The data of tide level and precipitation are both collected from LB hydrologic station covering the daily data from 1952 to 2009. The data are obtained from the Hydrological Administration of Fujian Province. The data quality is firmly controlled before its release. The consistency of data has been checked by the double-mass method which shows that all the data series used in this study are consistent. To ensure the representativeness of the precipitation data of LB station, we have checked the precipitation data from another station in Fuzhou, Chiqiao station, and found that the temporal statistics in the two stations are same. The tide level is measured using the Luo Zero Vertical Datum of China in this paper.

**Table 1:** Relationship of extreme values and their RPs for rainfall and tide level.

Maximum 24 h rainfall (mm)	Tide level (m)	RP (yr)
143.4	8.13	5
169.8	8.58	10
194.6	8.97	20
209.8	9.19	30
219.9	9.32	40
226.1	9.39	50

## Methods

### 3.1 Copulas

Copulas are a kind of distribution functions and have emerged as a powerful approach in simplifying multivariate stochastic analysis. According to Sklar’s theorem [36], to obtain a joint cumulative distribution function (CDF) 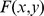 for random variables, *X* and *Y*, with marginal distributions 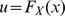 and 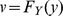, respectively, a copula function *C* makes.
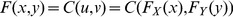
(1)


If 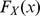 and 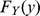 are continuous, then *C* is unique. Three widely used Archimedean copulas, Gumbel, Clayton, Frank copula are compared to select the best-fit one in this study. The expressions of the three copulas are introduced in Table 2. All of the three copulas are one-parameter copulas.

**Table 2:** Definition of the widely used one-parameter families of Archimedean Copulas.

Copula	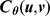	Parameter
Gumbel	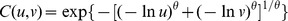	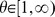
Frank	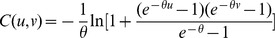	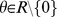
Clayton	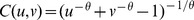	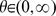

Identification of the copula is the next step when the parameters are estimated. The best-fit copula is selected in term of ordinary least squares (OLS) [37] criteria and Kolmogorov-Smirnov’s statistic D (K-S D) [38]. The copula with the minimum OLS value and passing the K–S test will be selected to build the joint distribution. The expression of OLS is:
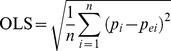
(2)where 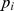 and 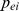 are the theoretic and empirical probabilities of the joint distribution, respectively.

### 3.2 Precipitation change detection

#### 3.2.1 Mann-Kendall change-point test

The non-parametric Mann-Kendall (MK) trend test [39], [40] is widely used in the detection of monotonic trends in a time series. The MK change-point test [41]–[43] is developed in term of MK trend test. For a time series of *n* observations under the null hypothesis *H*_0_ of no change, the MK statistics *S_k_* is defined as:
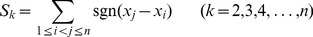
(3)


where
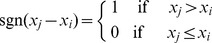
(4)


The mean and variance of *S_k_* are given by
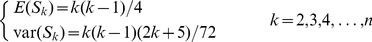
(5)


A two-sided significance test is employed to test the statistical significance of *S_k_* for this null hypothesis. Thus, define the statistic index *UF_k_* as:
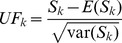
(6)


*UF_k_* is a normalized variable and a forward statistic sequence. The backward sequence *UB_k_* is also computed in term of the same equation but with a reversed series of data.

In the two sided test, the null hypothesis *H*_0_ is accepted or rejected according to whether the points in the forward sequence are outside the confidence interval (generally, with 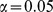). If there is any point outside the confidence interval, an increasing (*UF_k_* >0) or a decreasing (*UF_k_* <0) trend in the detection is indicated. The forward and backward curves of the test statistic, *UF_k_* and *UB_k_*, are plotted to localize the beginning of the change, at the intersection between the curves if it occurs within the confidence interval [44].

#### 3.2.2 Pettitt’s change-point test

Pettitt’s change-point test [45] detects a shift in the mean at any time, and calculates its statistical significance. For a time series of *n* observations, the Pettitt statistics 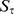 which makes a rank-based comparison between the observations located before and after a date *τ*, is calculated as
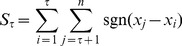
(7)


Where
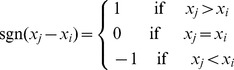
(8)


The change occurs at the time *T* for which 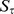 has the maximum absolute value *S*. The expressions of *T* and *S* are given by
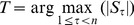
(9)




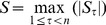
(10)


*S* is the final Pettitt statistics, and *T* is the date of change.

The associated probability used in significance testing is approximately estimated by:
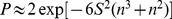
(11)


If it holds *P*<0.5, the change is significant.

## Results

### 4.1 Precipitation change point

**Figure 1:**
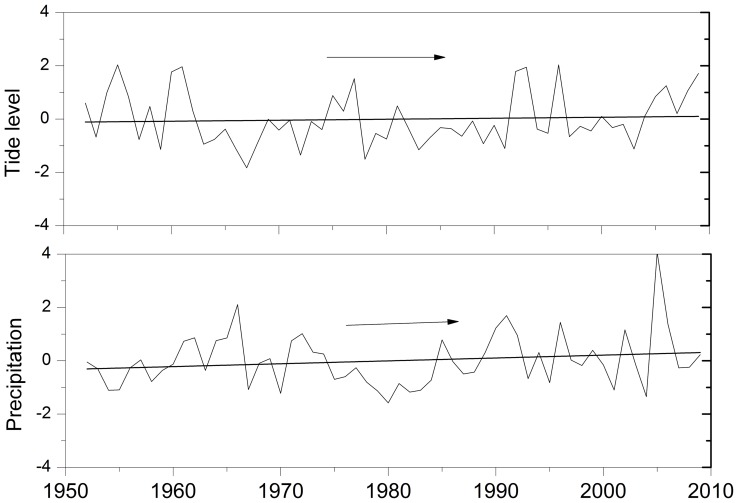
Trends of annual maximum 24-h precipitation and tide level.

The annual maximum 24-h precipitation series from 1952 to 2009 has a significantly increasing trend, and that of tide level is not much significant (Fig. 1). Therefore, only the change point of precipitation is detected in this paper. In Fig. 1, the variables are standardized using Z score transformation.

**Figure 2:**
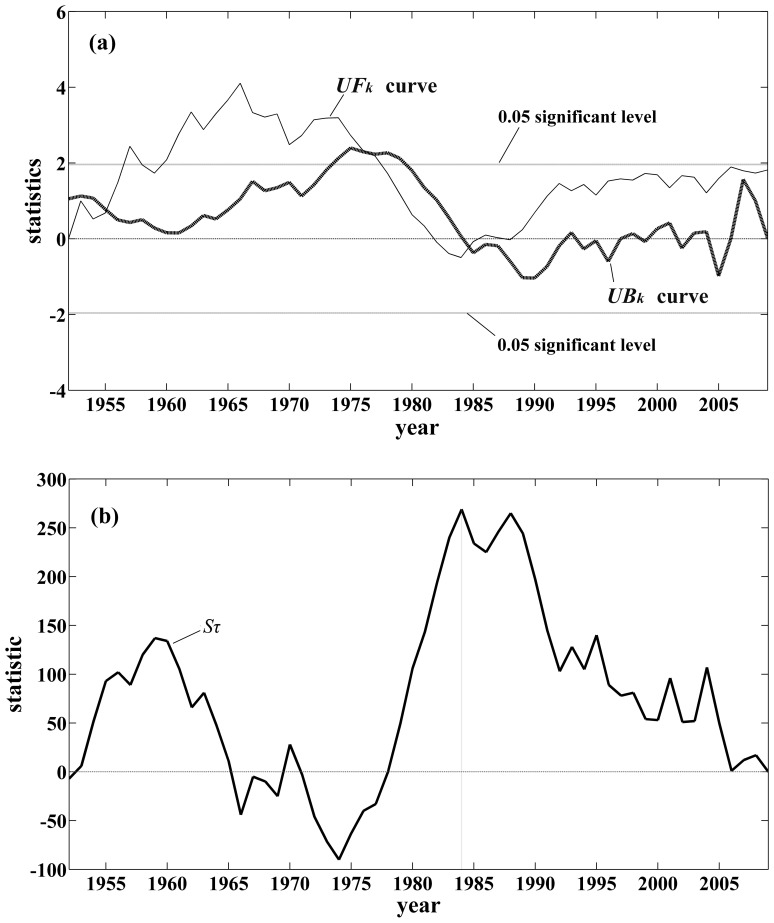
Precipitation change test in the annual maximum rainfall series from 1952 to 2009 in Fuzhou City: (a) Mann-Kendall test with the forward *UF_k_* (standard full line) and backward *UB_k_* (bold dash line) applications of the test; (b) Pettitt’s changing point test with the statistic 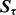. Vertical dot line represents the maximum value.

**Figure 3:**
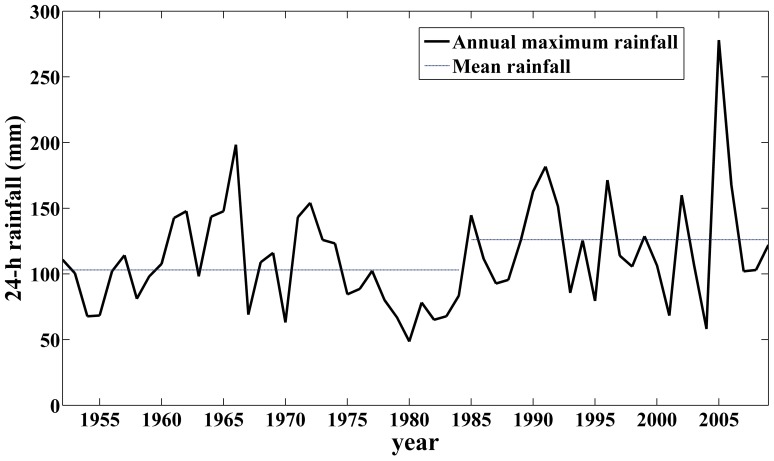
Annual maximum 24-h rainfall from 1952 to 2009 in Fuzhou City with the mean rainfall for each subsequence.

The change points of annual maximum 24-h precipitation in Fuzhou City are analyzed through the two change-point detection methods, MK and Pettitt’s tests. The MK test is firstly employed to detect the change points of the annual maximum 24-h precipitation over the period from 1952 to 2009. According to the test curve, there are two intersections, situated at the date of 1976 and 1984 (Fig. 2a). The intersection at the date of 1976 is outside the confidence interval at a significant level of 0.05. Conversely, the one at the date of 1984 is inside. Thus, it can be deduced that the change point is at the date of 1984. To ensure the location of the changing point, the Pettitt’s test is also employed to detect the annual maximum 24-h precipitation series. The statistic at the date of 1984 has the maximum value, illustrating that the change point is at this date (Fig. 2b), which is in agreement with the detection by the MK test. Thus, the annual maximum 24-h precipitation series could be separated into two subsequences: 1952–1984 and 1985–2009. The mean annual maximum 24-h precipitation is 103 mm in the first segment and that is 126 mm in the second segment (Fig. 3).

### 4.2 Copula-based probability analysis

#### 4.2.1 Marginal distributions

Pearson type-III (P-III) distribution is employed to fit the marginal distributions 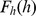 and 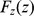 for *H* and Z in the two subsequences. The probability density function (PDF) and CDF of P-III distribution are:
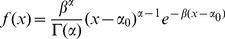
(12)




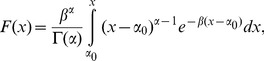
(13)Where 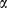, 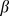 and 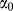 are the parameters. For *H* and *Z* in the two subsequences, the parameters estimated are shown in Table 3.

**Table 3:** Estimated parameters of P-III distribution for *H* and Z in the two subsequences.

Parameters	Rainfall *H*		Tide level *Z*	
	Sub 1	Sub 2	Sub 1	Sub 2
*α*	4.000	1.5055	4.5269	2.0120
*β*	0.0540	0.0231	3.0764	2.1629
*α*_0_	28.8314	64.2693	3.8486	4.5349

**Figure 4:**
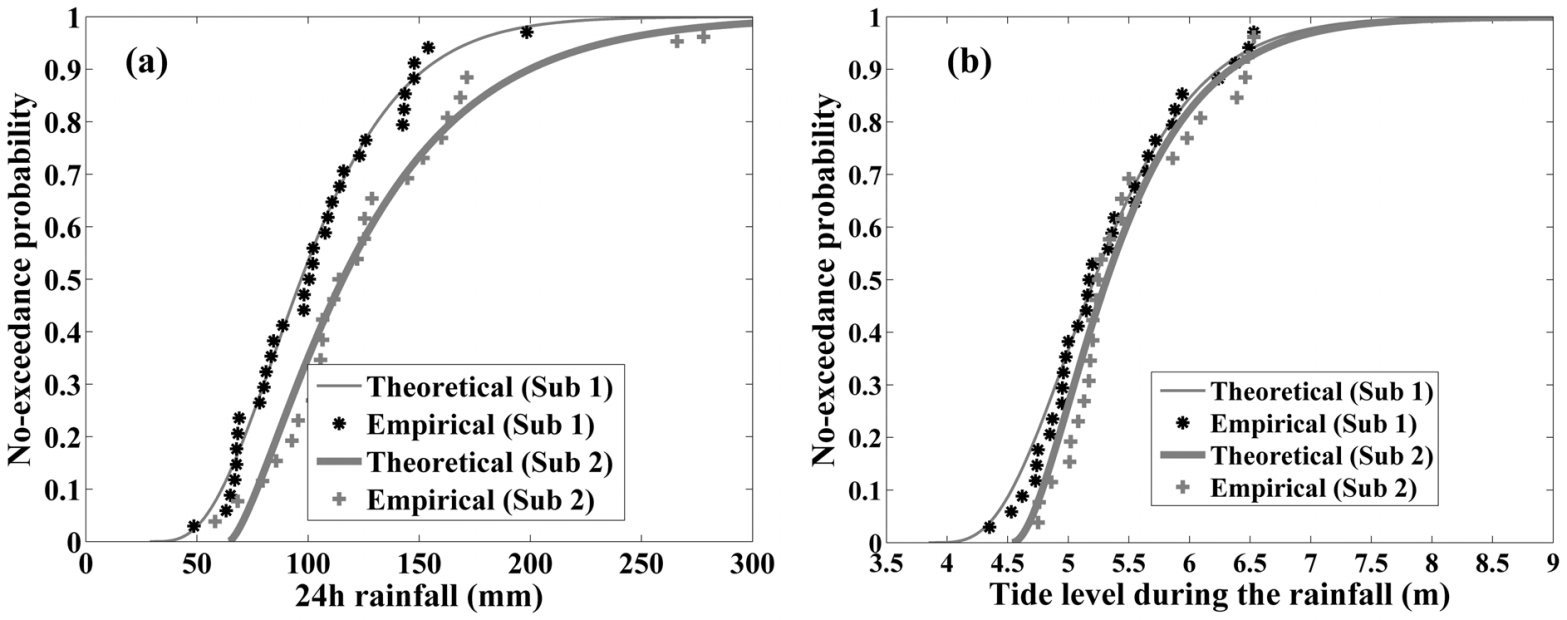
Comparisons of the empirical and theoretical marginal distributions in the two subsequences: (a) for 24-h rainfall; (b) for the tide level.

The marginal function can be estimated in term of the parameters in Table 3. The comparisons of the empirical and theoretical marginal distributions between the two subsequences for *H* and *Z* are plotted in Fig. 4, respectively. The marginal distribution can be acceptable by the K–S test with the significant level of 0.05.

Fig. 4a illustrates that the exceedance probability increases significantly due to precipitation change. For the precipitation of 150 mm, for example, the exceedance probability is 10.9% in subsequence 1. However, it is 26.7% in subsequence 2. It explains that the extreme precipitation has occurred more often in recent years in Fuzhou City to some extent. The storm tide during the extreme precipitation event also has increased after 1984, but not much significant (Fig. 4b).

#### 4.2.2 Copula selection

Maximum likelihood approach [14] is employed to estimate the parameters of copulas. The OLS value and K–S D of the three employed copulas, the Gumbel, Clayton, and Frank copulas, are estimated to choose the copula with the highest goodness-of-fit (Table 4). The threshold of K–S D with 95% confidence level is 0.231 for 33 statistical samples, and that is 0.264 for 25 samples. The maximum of K–S D testing the employed copulas for the two subsequences is 0.0896 in Table 4, smaller than the thresholds. So, each of the copula employed can pass the test. In the first segment, the OLS values of the Gumbel, Clayton, and Frank copulas are 0.0247, 0.0248, and 0.0250, respectively. Obviously, Gumbel copula has the minimum value of the OLS, illustrating that it is the best-fit copula to describe the probability properties of *H* and *Z* for the first segment. However, for the second segment, Frank copula has the lowest OLS value of 0.0273. That means Frank copula is the best choice to fit the joint distribution of *H* and *Z* for subsequence 2. It is interesting to notice that the copulas with the highest goodness-of-fit are different for the different subsequences. Thus, if precipitation change was not taken into account in the entire sequence analysis, the best-fit copula would be different from these in the different subsequences. That means precipitation change point has an impact on the selection of the joint CDF.

**Table 4:** Estimated parameters and goodness-of-fit test of copulas.

		Gumbel	Frank	Clayton
1952–1984 (Sub 1)	Parameter θ	1.0124	0.0651	0.0230
	OLS	***0.0247***	0.0248	0.0250
	K-S D	0.0688	0.0678	0.0688
1985–2009 (Sub 2)	Parameter θ	1.0714	0.6022	0.3360
	OLS	0.0325	***0.0273***	0.0290
	K-S D	0.0896	0.0870	0.0896

Equations (14) and (15) represent the joint CDF 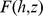 for each subsequence respectively with the 95% confidence interval of the parameter of the chosen copula. The expression of joint CDF for the first subsequence is
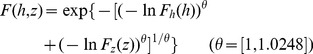
(14)


For the second subsequence the expression is
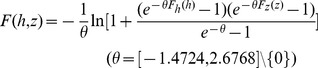
(15)


#### 4.2.3 Joint probability analysis

When the best-fit copula is chosen, we can estimate probabilities of the joint behavior of *H* and *Z* with some extreme values based on the joint CDF 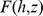. There are two probabilities to which we should pay much attention. One is the probability that both of the two variables, *H* and *Z*, exceed some RPs. This probability would determine the boundary conditions for the numerical modeling of the water body whose level is dependent on both precipitation and tide level. The other is the probability of *H* or *Z* over some extreme values. The flooding frequency would depend on this probability because each of the source, precipitation or storm tide, could cause flooding. For the two variables *H* and *Z* with their marginal distributions 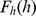 and 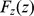
respectively, the two probabilities are given as:
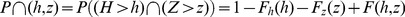
(16)




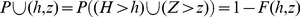
(17)


According to the Eqs. (14), (15), (16) and (17), Table 5 quantifies these two probabilities (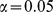) of precipitation and tide level in term of RPs.

**Table 5:** Probabilities of joint behavior of extreme rainfall and tide with different RPs.

24h rainfall		Storm tide		*P*∩(*h*,*z*) (*α* = 0.05)			*P*∪(*h*,*z*) (*α* = 0.05)		
*H* (mm)	*T_r_* (yr)	*Z* (m)	*T_t_* (yr)	Sub 1 (%/yr)	Sub 2 (%/yr)	Average increased Rate	Sub 1 (%/yr)	Sub 2 (%/yr)	Average increased Rate
143.4	5	8.13	5	[0.0359±0.0102]	[0.1366±0.0786]	280.42%	[13.704±0.0102]	[28.323±0.0786]	106.68%
169.8	10	8.13	5	[0.0198±0.0093]	[0.0887±0.0573]	349.03%	[5.6902±0.0093]	[16.901±0.0573]	197.02%
194.6	20	8.13	5	[0.0119±0.0077]	[0.0567±0.0387]	376.95%	[2.3881±0.0077]	[10.323±0.0387]	332.28%
226.1	50	8.13	5	[0.0065±0.0053]	[0.0306±0.0217]	369.93%	[0.8235±0.0053]	[5.4994±0.0217]	567.82%
143.4	5	8.58	10	[0.0139±0.0044]	[0.0576±0.0331]	314.75%	[13.606±0.0044]	[28.182±0.0331]	107.13%
169.8	10	8.58	10	[0.0080±0.0041]	[0.0374±0.0242]	367.53%	[5.5820±0.0041]	[16.733±0.0242]	199.76%
194.6	20	8.58	10	[0.0051±0.0036]	[0.0239±0.0164]	366.07%	[2.2749±0.0036]	[10.136±0.0164]	345.57%
226.1	50	8.58	10	[0.0031±0.0027]	[0.0129±0.0092]	311.23%	[0.7069±0.0027]	[5.2971±0.0092]	649.38%
143.4	5	8.97	20	[0.0052±0.0018]	[0.0252±0.0145]	385.41%	[13.570±0.0018]	[28.125±0.0145]	107.26%
169.8	10	8.97	20	[0.0031±0.0017]	[0.0164±0.0106]	425.22%	[5.5419±0.0017]	[16.664±0.0106]	200.69%
194.6	20	8.97	20	[0.0021±0.0016]	[0.0105±0.0072]	397.08%	[2.2329±0.0016]	[10.060±0.0072]	350.52%
226.1	50	8.97	20	[0.0014±0.0012]	[0.0057±0.0040]	303.36%	[0.6636±0.0012]	[5.2143±0.0040]	685.77%
143.4	5	9.39	50	[0.0019±0.0007]	[0.0108±0.0062]	453.89%	[13.557±0.0007]	[28.099±0.0062]	107.27%
169.8	10	9.39	50	[0.0012±0.0007]	[0.0070±0.0045]	478.65%	[5.5278±0.0007]	[16.633±0.0045]	200.90%
194.6	20	9.39	50	[0.0009±0.0007]	[0.0045±0.0031]	425.53%	[2.2181±0.0007]	[10.026±0.0031]	351.98%
226.1	50	9.39	50	[0.0006±0.0005]	[0.0024±0.0017]	301.20%	[0.6484±0.0005]	[5.1776±0.0017]	698.52%

*H*: annual maximum 24-h rainfall; *T_r_*: RP of *H*; *Z*: highest tide level during the day of *H*; *T_t_*: RP of *Z*.

**Figure 5:**
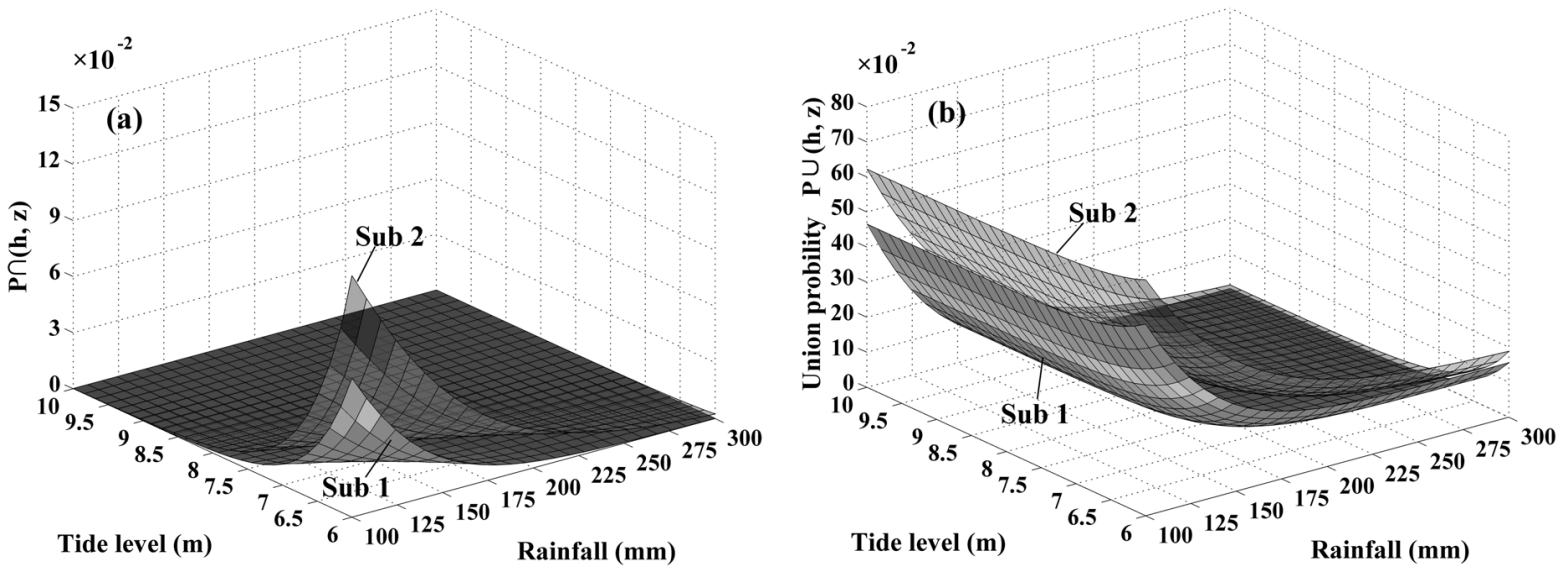
Comparison of probability between subsequence 1 and 2 in term of absolute values: (a) probability of 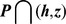; (b) probability of 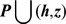.

**Figure 6:**
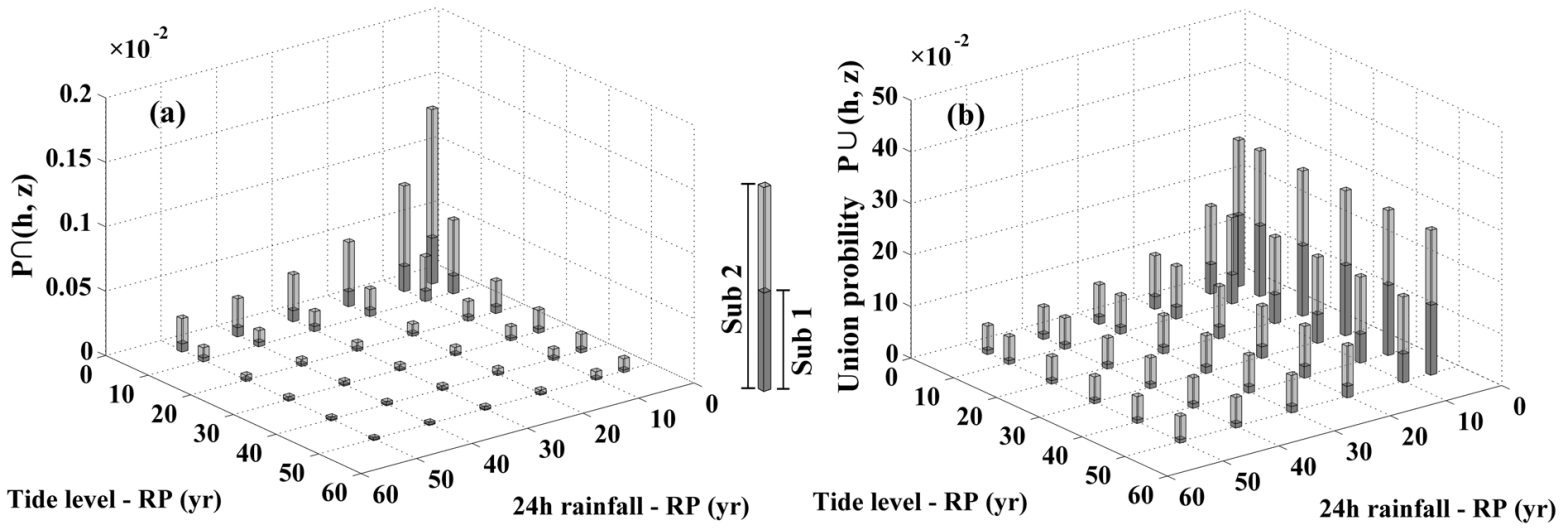
Comparison of probability between subsequence 1 and 2 in term of RPs: (a) probability of 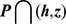; (b) probability of 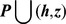.

To intuitively illustrate the changes between the two subsequences, the joint probabilities on average of precipitation and tide level are also plotted in Figs. 5 and 6 in different form. For the joint behavior of the precipitation and storm tide from 5 to 50 RPs in Table 5, the probability of 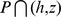 of a 5-year RP precipitation and 5-year RP storm tide has the maximum medium value of 0.04% in subsequence 1, and that of 0.14% in subsequence 2. The chance that extreme precipitation and storm tide happen simultaneously is small in the first subsequence. However, that has increased significantly in the second subsequence (Fig. 6a). The average increased rate of this chance is up to 369.14%. The maximum medium value of union probability of 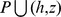 is 13.70% at the joint behavior of a 5-year RP precipitation and 5-year RP storm tide in subsequence 1, and that is 28.32% in subsequence 2. There is a great increase of 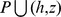 after 1984 (Fig. 6b). The mean probability increases 325.53% in the second subsequence as compared to that in subsequence 1. It is obviously concluded that the joint probabilities have risen more than 300% after 1984 as compared to that of the past in Fuzhou City.

### 4.3 Joint RP of precipitation and tide for flooding preparedness

As mentioned before, flooding would happen when precipitation or tide level exceeds the design standard. Thus, the design joint RP (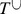) for flooding preparedness in a coastal area can be defined by either precipitation or the tide level over design standards (i.e. 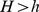 or 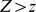). The expression of this joint RP in a coastal city is:
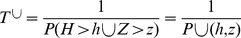
(18)


**Figure 7:**
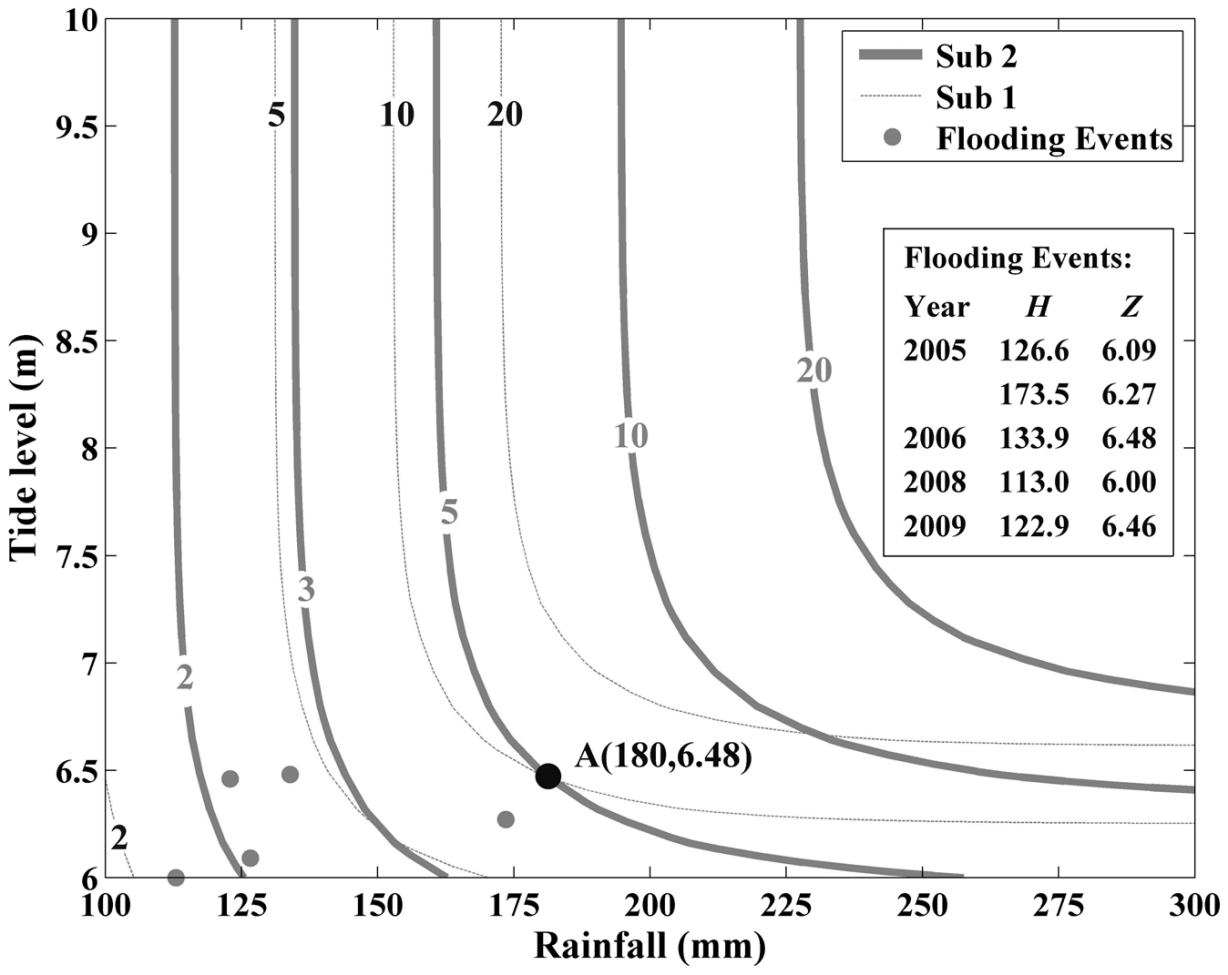
Contours of the design joint RP for flooding preparedness for each subsequence.

Accordingly, the contours of 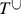 are plotted in Fig. 7. For a same combination {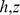}, 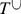 in subsequence 2 is smaller than that in subsequence 1, showing that the joint behavior of extreme precipitation and storm tide has occurred more often over the past 25 years. For example, for the black point A in the Fig. 7, the combination of precipitation of 180 mm and storm tide of 6.48 m, 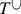 is 10 years in subsequence 1. However, it is 5 years in subsequence 2. It implies that as the environment changing, the preparedness measures for flooding which are adequate 10 or 20 years will not seem so adequate in the future. For Fuzhou city, the frequency of flooding and the cost from flooding would be rising. In fact, in recent years, the flooding has occurred almost every year from 2005, with more than one time in 2005 and 2011. Due to the lack of data on severe flooding events, only 6 years of characteristic variables of severe flooding events with precipitation exceeding 100 mm and tide level over 6 m from 2005 to 2010 are collected and plotted in Fig. 7. The time period of 6 years is relatively short. However, it can demonstrate flooding have occurred more frequently to some extent, which is consistent with the experience of local residents. The three events with similar characteristics in which the precipitation is between 120 mm and 135 mm happened 3 times in 2005, 2006 and 2009. In other words, the RP of a severe flooding event with precipitation and tide level at this range is nearly 2 years. That is closed to the RP plotted in Fig. 7. Considering the lack of the flooding data in this paper, the next step is to extend the time series of severe flooding events to make the verification rigorous.

## Discussions and Conclusions

In the last decade, flooding has occurred more often than before in most cities in China, especially in coastal cities. In the traditional design of drainage facilities the impact of storm tide on flooding is ignored or underestimated in coastal areas, such as Fuzhou, Haikou. Besides, the environment change including climate changes and rapid urbanization may be not considered in the design of drainage systems. Thus, the drainage system of today is outdated and not adequate to satisfy the demand of flooding preparedness, which leads to the increase of flooding frequency and losses. It is therefore urgent to develop a new design standard and preparedness that can adapt with the changing environment to address this problem. The work in this paper gives a perspective to understand the increased flooding risk and to help address the problem of more frequent flooding in China.

We investigate the joint probability of extreme precipitation and storm tide using a copula-based model taking the precipitation change into consideration in a coastal city, Fuzhou City. The precipitation change is detected by the M-K and pettitt’s tests. Higher occurrence of extreme precipitation events can be detected after 1984 in term of the two tests. Similar significantly increasing trends after the mid-1980s are found in some parts of China, such as the Pearl River basin [46] and the Yangtze River Delta [47]. The date of mid-1980s is almost consistent with the moment of rapid urbanization in China [48].

The joint distribution of precipitation and storm tide is established through the best-fit copula for different subsequences. The precipitation change has an impact on copula selection. For the first subsequence, Gumbel copula is the best-fit copula. However, Frank copula is the best one for the second subsequence. The joint probability has significantly increased by more than 300% on average after 1984. The estimation quantitatively illustrates that flood risk in Fuzhou city has significantly increased and flooding is supposed to happen more often in recent years. This is consistent with actual occurrences.

For a same combination {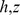}, the design joint RP of them for flooding preparedness in subsequence 2 is smaller than that in subsequence 1. It can be concluded that with environment changing, the preparedness measures for flooding which are adequate 10 or 20 years would not seem so adequate any more in the future. The flooding would occur more often if no defense enhanced in Fuzhou City and the cost from flooding may be rising in the future.

This paper demonstrates that it is necessary to consider the joint probability of precipitation and tide level when determining the design standard for flood preparedness. The sea level which closely relates to the tide level would be significantly rising in the future [49]. So the prediction of the sea level rise should be analyzed in the development of the future design standard of flood preparedness. It would be the topic of our future work.
